# Evaluation of simulation models to mimic the distortions introduced into squiggles by nanopore sequencers and segmentation algorithms

**DOI:** 10.1371/journal.pone.0219495

**Published:** 2019-07-18

**Authors:** Michael Smith, Rachel Chan, Paul Gordon

**Affiliations:** 1 Department of Electrical and Computer Engineering, University of Calgary, Calgary, Alberta, Canada; 2 Department of Radiology, University of Calgary, Calgary, Alberta, Canada; 3 Department of Health Genomics and Informatics, University of Calgary, Calgary, Alberta, Canada; 4 Alberta Childrens’ Hospital Research Institute, Calgary, Alberta, Canada; Mayo Clinic Arizona, UNITED STATES

## Abstract

Nucleotides ratcheted through the biomolecular pores of nanopore sequencers generate raw picoamperage currents, which are segmented into step-current level signals representing the nucleotide sequence. These ‘squiggles’ are a noisy, distorted representation of the underlying true stepped current levels due to experimental and algorithmic factors. We were interested in developing a simulation model to support a white-box approach to identify common distortions, rather than relying on commonly used black box neural network techniques for basecalling nanopore signals. Dynamic time warped-space averaging *(DTWA)* techniques can generate a consensus from multiple noisy signals without introducing key feature distortions that occur with standard averaging. As a preprocessing tool, *DTWA* could provide cleaner and more accurate current signals for direct *RNA* or *DNA* analysis tools. However, *DTWA* approaches need modification to take advantage of the *a-priori* knowledge regarding a common, underlying gold-standard *RNA / DNA* sequence. Using experimental data, we derive a simulation model to provide known squiggle distortion signals to assist in validating the performance of analysis tools such as *DTWA*. Simulation models were evaluated by comparing mocked and experimental squiggle characteristics from one *Enolase* mRNA squiggle group produced by an Oxford MinION nanopore sequencer, and cross-validated using other *Enolase*, *Sequin R1_71_1* and *Sequin R2_55_3* mRNA studies. New techniques identified high inserted but low deleted base rates, generating consistent *x1*.*7* squiggle event to base called ratios. Similar probability density and cumulative distribution functions, *PDF* and *CDF*, were found across all studies. Experimental *PDFs* were not the normal distributions expected if squiggle distortion arose from segmentation algorithm artefacts, or through individual nucleotides randomly interacting with individual nanopores. Matching experimental and mocked *CDFs* required the assumption that there are unique features associated with individual raw-current data streams. *Z-*normalized signal-to-noise ratios suggest intrinsic sensor limitations being responsible for half the gold standard and noisy squiggle *DTW* differences.

## Introduction

Raw current signals (nanostreams) are recorded as each DNA or RNA molecule is ratcheted one nucleotide at a time by a motor protein through a nanopore biomolecule in a sensor array on a device such as the Oxford Nanopore MinION sequencer [1, 2]. Various techniques can be used to convert the picoamperage raw signal (e.g. 4000Hz on the MinION) into a series of stepped current levels corresponding ideally 1:1 to the ratcheted nucleotides [3]. However, in practice the series of stepped current levels (a.k.a. “squiggles” [4]) are a stretched and distorted representation of the DNA or RNA sequence.

The original equipment manufacturer (OEM) provides a k-mer model for the correspondence of nucleotide sequence to current levels, based on many experiments done internally. This allows us to trivially calculate a ‘golden’ signal for any given nucleotide sequence. Raw signal segmentation algorithms generate squiggles that are a noisy and distorted representation of the underlying true stepped current levels due many factors. These include 1) the uneven production of current steps per unit time due to the stochastic nature of the motor protein driving the steps, 2) homopolymerism where long chains of multiple identical bases are misinterpreted, 3) in-silico chimeric reads, 4) experimental sensor errors and noise generated measuring the current by steric configuration of the nucleotides and 5) segmentation artefacts. These errors can be represented as a certain probability of insertions and deletion into the squiggle. These respectively represent signals falsely interpreted as the presence of additional bases, or the failure of the passage of a base through a nanopore to generate a raw signal that can be segmented into a squiggle event.

**Fig 1:**
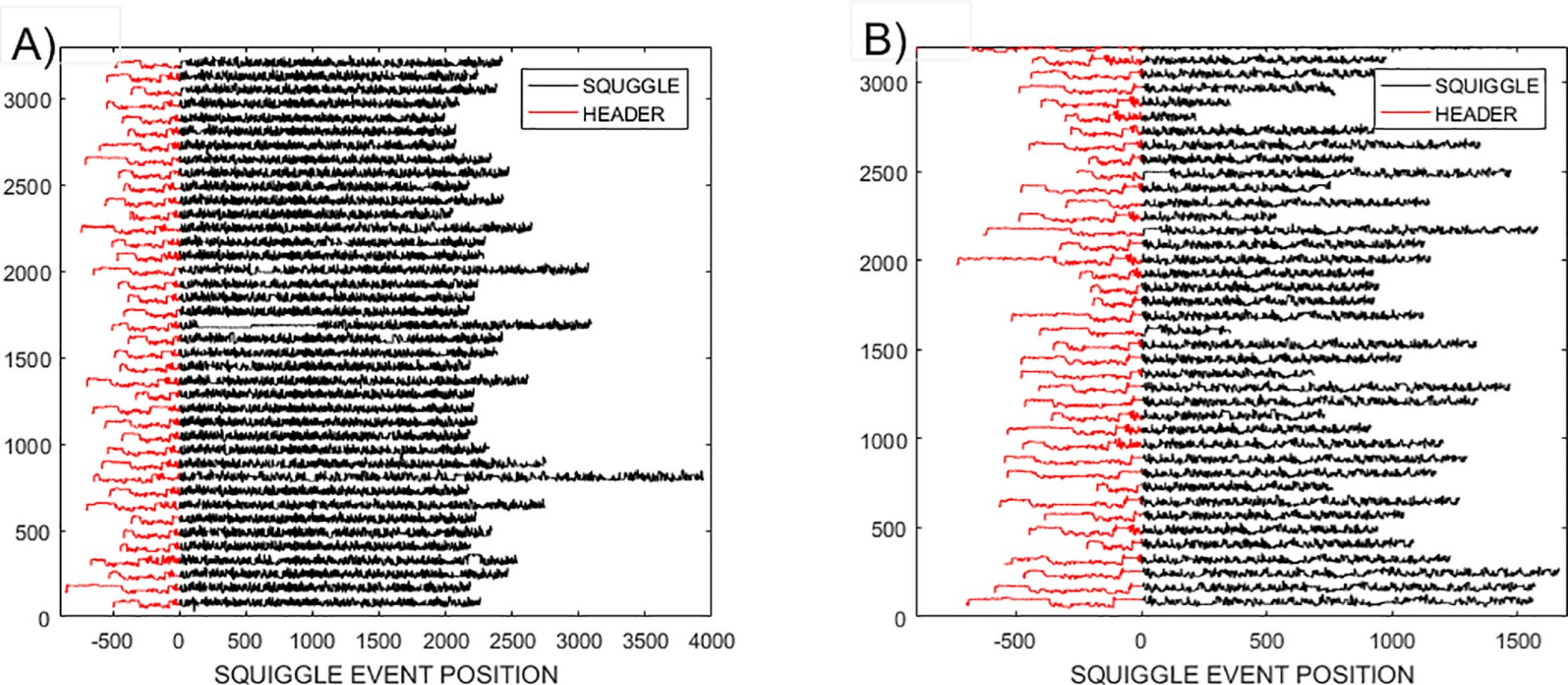
Thirty representative squiggles from the A) Enolase and B) Sequin R2-55-3 studies. The leaders, shown in red, can be identified by differences between local leader and global stream characteristics of intensity means and standard deviations. A further data pruning was used to remove stream outliers that varied by more than 5 standard deviations from the mean ensemble characteristics.

The difference between the squiggles, (see Fig 1), and the underlying gold standard that describes *RNA / DNA* molecular characteristics can be identified through dynamic time warping (*DTW*). This provides a comparison where the similarity between a pair of squiggles is maximized by stretching each squiggle while minimizing the total Euclidian distance between them [5]. Chan *et al*. [6] demonstrate that using the *DTW* Barycentre averaging, *DBA*, algorithm [7, 8] could generate a consensus from multiple noisy squiggles, which cannot be achieved through the direct use of *DTW* which only compares signal pairs. *DBA* is able to generate a consensus signal without averaging out the key-characteristics of individual data-sets as would occur with standard averaging approaches. It was suggested that the generation of a clean consensus signal would be a useful preprocessing stage leading to more accurate results following the application of existing analysis tools.

Our experiments comparing the *DBA* consensus signal [7] with those generated by two other dynamic time warped-space averaging, *DTWA*, algorithms proposed by Schultz and Jain [9] have identified a major difference between the uses of *DTWA* algorithms in other fields compared to this new squiggle-space application. *DTWA* algorithms are commonly applied to generate a consensus signal from similar signals from multiple different sources. *DTWA* algorithms have not been optimized to take advantage of unique *a-priori* information available in this new signal space environment. Each squiggle is formed from stepped current signals segmented from raw current nanostream signals convergent towards the same fundamental gold-standard sequence of DNA or RNA molecules.

We propose the development of a novel simulation framework that provides a detailed characterization of the distortions introduced by nanopore sequencing devices or in conjunction with associated segmentation methods. This framework can be used to assist in optimizing *DTWA* algorithms to make use of the domain-specific gold-standard not available in other fields of study. Both *DTWA* and the proposed simulation model are intended as tools to support a white-box approach to identify common distortions, rather than relying on commonly used black box neural network techniques for basecalling nanopore signals.

This paper is organized as follow. The Methods section outlines techniques used to identify, and quantify, the key distortions present in squiggles. A simulation model incorporating these characteristics is proposed. The Result section provides details of an empirical investigation of the key distortion parameters identified as present in *Enolase* squiggles generated by the Oxford MinION sequencers. An investigation is undertaken on how to use these noisy parameter estimates within a simulation model to generate mocked squiggles whose characteristics best match those of actual squiggles. Preliminary results of applying this proposed model to the smaller and noisier *Sequin R1_71_1* and *Sequin R2_55_3* RNA [10] squiggle ensembles are provided. The Conclusion section summarizes our findings and proposed future research directions.

## Methods

This section proposes techniques to identify the key distortions present in the squiggle data ensemble. First, cleaning is performed to remove all streams that are extreme outliers from the data ensemble averages. The probability density function, *PDF*, and the cumulative distribution function, *CDF*, are then determined. Potential tools are proposed to estimate the number of insertion and deletion distortions in a given squiggle relative to the calculated ‘golden’ squiggle associated with the underlying nucleotides spiked into the experiments. Insertions and deletions are respectively an indication of bases erroneously indicated as present or absent from a squiggle without attribution of the source of the error. Finally, a procedure to estimate the noise level present in experimental squiggles is detailed.

### Generation and cleaning of noisy squiggles

Data were generated using the SQK-RNA001 direct RNA sequencing kit and protocol v1.08 with the MinION Mk1B sequencer [1, 2] running the OEM MinKnow device control software. One DNA sample contained only the OEM-provided yeast enolase mRNA spike-in. Two others, *R1_71_1* and *R2_55_3*, were supplemented with RNA Sequin v1 Pool A [10].

Squiggles whose distortions were outliers from the average characteristics of the approximately 7000 squiggles from the *Enolase* ensemble were eliminated using the following procedure.

- It was found experimentally that the squiggles segmented from all three raw current streams had a mean length *x1*.*7* larger than that of the underlying gold standard squiggle. All squiggles whose length was smaller than the gold-standard’s length were therefore deleted as gross outliers. This left signals similar to those shown in Fig 1A and 1B for the *Enolase* ensemble and the noisier *R2_55_3* data ensembles respectively.
- The leaders of the streams, shown in red, are associated with the enzyme used in the wet-lab process to guide the *RNA* to the sensor. While physically essential to the process to generate a raw signal, this leader does not contribute directly to the gene sequence of interest. The header was identified by its local mean and standard deviations not matching those of the general stream.
- The remaining squiggle lengths did not follow a normal distribution, making it inappropriate to reject outliers identified on the basis of characteristics deviating by more than two standard deviations from various ensemble squiggle mean values. We therefore performed a less stringent pruning and only removed outliers whose intensity, mean and length characteristics deviated by more than 5 standard deviations from the ensemble global and local means.
- To identify in-silico chimeric reads, we broke each squiggle into sections and calculated the mean of the standard deviations of section event intensities. Squiggles were rejected if this mean was more than 2 standard deviations from the ensemble mean of standard deviations of section intensities.

### Statistical characterization of sequences

#### Investigation of overall squiggle length statistics

In their preliminary investigation into using *DBA* to generate consensus signals, Chan *et al*. [6] proposed a simple distortion model involving equal probabilities of base information in the calculated gold standard squiggle appearing duplicated or absent, i.e. deleted, in a given segmented squiggle. However, we have identified that both the *Enolase* and *Sequin R1* distorted squiggles were typically *x1*.*7* times than their respective gold standard length, indicating significantly higher insertion probabilities compared to deletion probabilities. To better characterize the squiggle distortions, the length details of the cleaned *Enolase* experimental squiggles were identified. The experimental probability density function, *E-PDF*, was then determined to allow calculation of the experimental cumulative distribution function, *E-CDF*. These density functions can be used to evaluate the accuracy of the mocked probability density, *M-PDF*, and cumulative distribution. *M-CDF*, functions generated by the proposed simulation models.

### Estimating the overall Signal-to-Noise statistics

The simulation model proposed by Chan *et al*. [6] assumed that Gaussian white noise would be present with a standard deviation of between 2% and 4% of the maximum range of gold-standard intensities. These correspond to signal-to-noise ratios, *SNR*, of 50 : 1 and 25 : 1 respectively using a standard *SNR* definition of

SNR=Maximumrangeofsignalintensities/standarddeviationofnoise (1)

*DTW* and *DTWA* studies are typically undertaken on z-normalized versions of the signal,

*S*_*Z-NORM*_*(n) = (S(n)–S*_*MEAN*_*) / stdev(S(n)*, which removes the signal mean, *S*_*MEAN*_, before normalizing the signal amplitude to a standard deviation, *stdev*, of 1. As this format does not characterize signals in terms of a range of intensities, we have proposed a new *SNR*_*Z-NORM*_ measure in our research into noise characterization of signals in squiggle space:

$$SNR_{Z-NORM}=stdev\left(S_{Z-NORM}\left(n\right)\right)/stdev\left(noise\right)=1/stdev\left(noise\right)$$ (2)

We used the following procedure to generate an estimate of the experimental *SNR*_*Z-NORM*_:

- The *Enolase* ensemble contains squiggles of lengths ranging from 1400–3500. The 60 squiggles in the length range 1600–1700 were selected to provide a dataset with fairly homogeneous insertion, deletion and noise characteristics across all members of this sub-ensemble.
- A *DTW* comparison was made between pairs *P* and *Q* of this squiggle subset:
  $$\left[distDTW_{P\_Q},WP_{P}(n),WP_{Q}(n)]=dtw(Squiggle_{P}(n),Squiggle_{Q}(n)\right)$$ (3)
  to identify the *WP*_*P*_*(n)* and *WP*_*Q*_*(n)* warping paths with minimal Euclidean distance.

- The difference, *DIFF*_*P-Q*_, between the warped versions of the squiggles
  $$DIFF_{P-Q}(n)=Squiggle_{P}(WP_{P}(n))-Squiggle_{Q}(WP_{Q}(n))$$ (4)
  will be predominately noise if we assume that the squiggles with similar lengths have similar characteristics. This provides an estimate of the experimental *SNR*_*Z-NORM*_ as
  $$SNR_{Z-NORM}(n)=K\;stdev(S_{Z-NORM}(n))/(stdev(DIFF_{P-Q}(n))/\sqrt{2})$$ (5)

The mean and standard deviation of *S*_*Z-NORM*_ were determined. The scaling factor *K* accounts for any contribution to *DIFF*_*P-Q*_ from differences between the locations of specific insertions and deletions across two similar streams. The statistics of comparing two noisy sequences requires the introduction of the *root(2)* term.

#### Proxies to evaluate overall insertion and duplication statistics

To identify the characteristics of the duplications and deletions introduced into segmented current steps, we have adopted a procedure that uses *DTW-*based proxies to compare the squiggle associated with the calculated gold-standard and the ensemble of experimental noisy squiggles. An accurate simulation model will lead to these proxies providing similar results for both experimental and mocked squiggles, even when the proxies are not entirely appropriate for determining actual insertion and deletion rates.

The *DTW* algorithm [11, 12] returns the warping paths, *WP*_*GOLD*_*(n)* and *WP*_*SQUIGGLE*_*(n)* necessary to minimize the Euclidian distance, *distDTW*, between the known gold standard and the *n*^*th*^ squiggle

$$\left[distDTW_{n},WP_{GOLD}(n),WP_{SQUIGGLE}(n)]=dtw(gold,squiggle(n))\right)$$ (6)

The presence of *ME* multiple entries in the warping path *WP*_*GOLD*_*(n)* implies that the gold standard stream must be time-stretched at *ME* locations to match this squiggle. We propose that *ME* be interpreted as an estimate of the number of duplications (a.k.a. insertions), *ME*_*DUPLICATIONS*_, present in the squiggle and not present in the gold stream. In an equivalent manner, the multiple entries in the *WP*_*SQUIGGLE*_*(n)* warping path can be interpreted as indication of *ME*_*DELETIONS*_ entries in the gold standard stream that are absent in the squiggle. The experimental mean and standard deviations for single and multiple *ME*_*DUPLICATIONS*_ and *ME*_*DELETIONS*_ were calculated for the squiggle ensemble as a whole, and for small groups of squiggles with similar lengths.

#### Proposed simulation model

**Fig 2:**
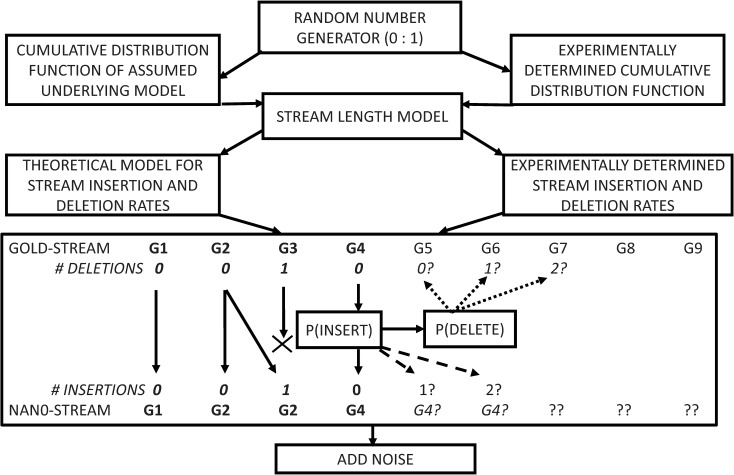
The proposed simulation model uses either an experimentally determined or proposed cumulative distribution function to generate a stream length model for the mocked squiggle. Each gold-stream base is either inserted into, or deleted from, the mocked stream based on probability functions determined experimentally or from an assumed theoretical model.

The simulation model proposed in Chan et al. [6] assumed that there was an *X%* chance of both deletions and insertions, and *(100 – 2X)*% chance of an undistorted squiggle value. The schematic of a more complex simulation model better matched to the experimental squiggle characteristics is shown in Fig 2.

- A random number generator selects a stream length model based on values from a cumulative distribution function, *CDF*, determined directly from the experimental data. Alternatively, as discussed later, *CDFs* can be calculated from proposed theoretical distribution models that approximate the experimental data.
- Gold-standard squiggle event values are singly copied, or duplicated, into the mock squiggle based on the probability *P(INSERT)* determined experimentally or from a proposed duplication model.
- The experimental or theoretical probability *P(DELETE)* determines whether the next gold-stream entry will be used to generate a mocked-squiggle value or whether multiple entries in the gold-squiggle will be skipped over, i.e. deleted from the mocked-squiggle.
- Finally, to model the level of experimental noise on a squiggle, Gaussian white noise was added until the mocked signal’s *SNR*_*Z*−*NORM*_ matched the experimental *SNR*.

## Results

The empirical *Enolase* squiggle ensemble was broken into groups of 2000 to allow evaluation of its anticipated temporal characteristics due to factors that may change later in an experiment, e.g.motor fuel and voltage bias levels. Basic squiggle length, insertion and deletion rates and noise characteristics were evaluated for each group. Several simulation models were proposed for one temporal grouping, and initially accepted based on whether their mocked cumulative distribution function, *M-CDF*, matched the experimental *Enolase E-CDF* of that group. Models were then rejected based on whether their mocked squiggle characteristics matched the observed insertion and deletion rates. Finally, the experimental *E-CDF* and best model’s *M-CDF* for this initial group were compared to the *E-CDF* and *M-CDF*s of other real and mocked groupings of *Enolase* squiggles, and for the *Sequin R1_71_1* (116 squiggles) and *Sequin R2_55_3* (122 squiggles).

### Determination of squiggle characteristics

#### Data length characteristics

**Fig 3:**
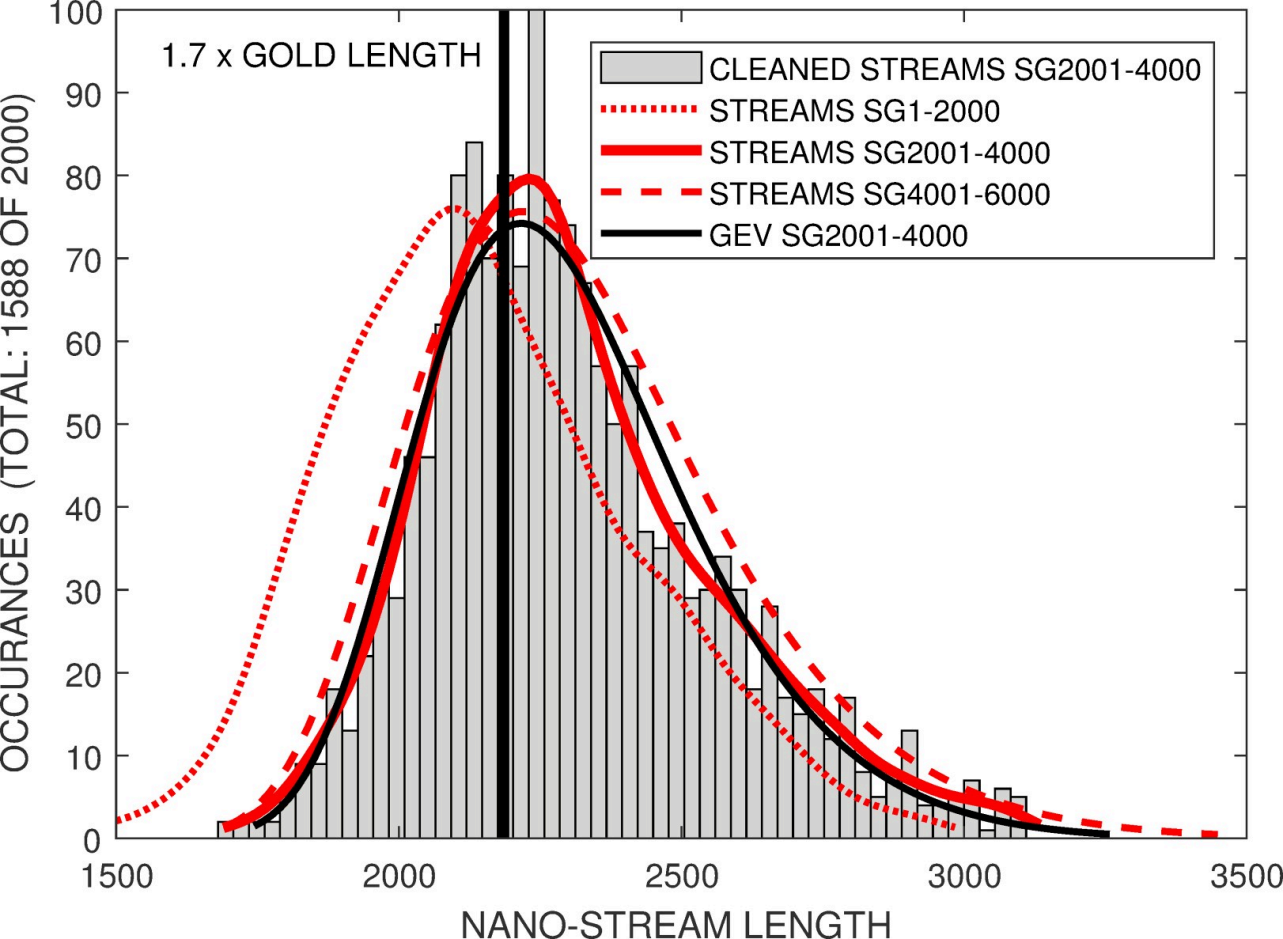
The original length distribution from *SG2001-4000* (red line) has a broad *PDF* peak at around *x1*.*7* longer than the gold standard length, with a long high-length tail. Other stream groupings (dotted and dashed red lines) have similar, but shifted, *PDF*’s. The *GEV PDF* for *SG2001-4000* (solid black line) is shown for comparision.

Typically 80% of squiggles in a cross-validation group remained after removal of the squiggles with characteristics that were gross outliers from the ensemble’s intensity mean and standard deviation and stream length, and the removal of squiggles with in-silico chimeric reads. Fig 3 compares the histograms of the cleaned experimental squiggle lengths from three groupings of 2000 *Enolase* squiggles.

The smoothed probability density function, *E-PDF* for the first temporal group, SG1-2000, had a broad peak at *x1*.*6* of the gold standard length. The second (SG2001-4000, solid red line) and third (SG4001-6000) temporal groupings had similar broad peaks at *x1*.*70* and *x1*.*69* times the gold standard length respectively.

It is to be expected that the experimental length distortion measure evaluated as squiggle events divided by bases called, would be nearly identical amongst the *Enolase* squiggle groupings. The samples had been prepared in the same biological manner with raw current signals generated by the same nanopores. It might be anticipated that similar levels of segmentation artefacts would be generated from their nearly identical features. However, it was not anticipated that the *Sequin* samples prepared in a different, i.e. synthetic, manner and analyzed using raw current signals generated from a different nanopore device would produce such similar *x1*.*7* squiggle event to bases called ratios. Additionally, the sequence composition of the Sequins and Enolase are very different, yet generate similar distortion levels. Later in this paper, we offer explanations for why we believe this common ratio seen across multiple ensembles is not an artefact associated with an imperfect segmentation algorithm.

The *E-PDF* did not exhibit a normal distribution, having a significantly longer tail above the peak than below. We empirically attempted to fit the *E-PDF* to non-Gaussian distributions. The black-line in Fig 3 shows the generalized extreme value (*gev*) distribution [13] fitted to the *SG2001-4000* group of squiggles. Neither this nor the *log-normal* [14] or *log-logistic* [15] long-tailed distributions investigated provide a close match for both the *E-PDF*’s broad intensity peak and tails. However, their approximate fit allows their distribution parameters to be used as a practical proxy for evaluating similarity between the *Enolase E-PDF* and *M-PDF*.

**Fig 4:**
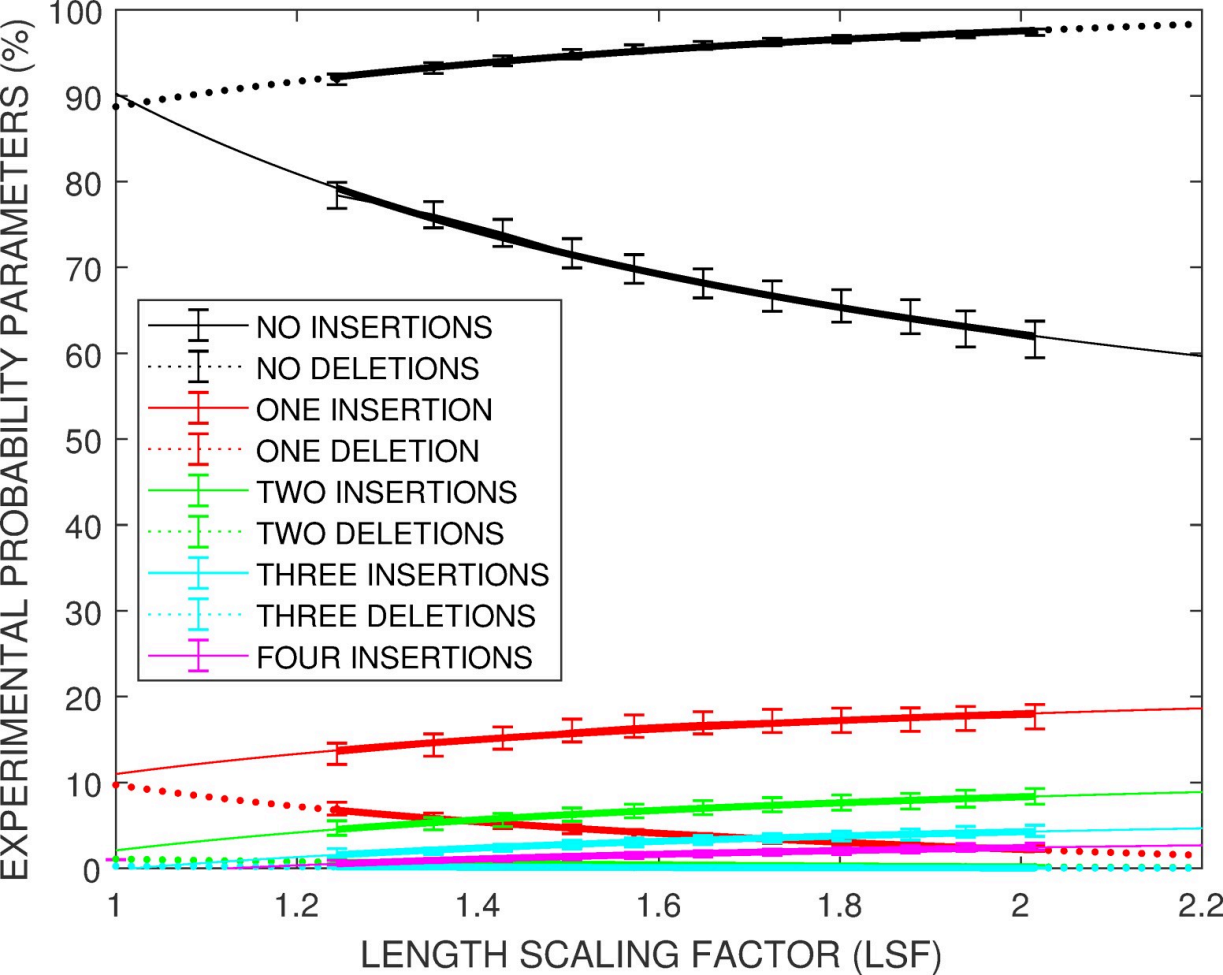
Experimental insertion and delete rates can be characterized by expressions of the form *A + B (1–1 / LSF)* where the length scaling factor *LSF = squiggle_length / gold_standard_length*.

The experimental insertion and deletion rates from groups of *Enolase* squiggles differing by no more than *50* points in their average length are displayed in Fig 4. Empirically it was found that both the *P(INSERT)* and *P(DELETE)* probabilities can be described by experimental curves of the form

P(LSF)=A+B(1–1/LSF) (7)

where the length scaling factor, *LSF*, is given by

LSF=squigglelength/gold−standardlength (8)

The *A* and *B* parameters for the various insertion and deletion rates are shown in Table 1. The full 7000 stream *Enolase* study was analyzed when generating Fig 4 in order to provide estimates of *P(LSF)* in the lowly populated tails of the data.

**Table 1:** The experimentally determined insertion and deletion probabilities for a distorted squiggle’s length scaling factor (*LSF*) can be modelled as *A*+*B*(1+1/*LSF*).

	A	B
**NO INSERTIONS**	90.2	-56.1
**NO DELETIONS**	88.7	17.7
**ONE INSERTION**	11.0	14.0
**ONE DELETION**	9.7	-15.0
**TWO INSERTIONS**	2.1	12.5
**TWO DELETIONS**	1.1	-1.8
**THREE INSERTIONS**	-0.2	8.8
**THREE DELETIONS**	0.3	-0.4
**FOUR INSERTIONS**	-0.7	6.2

Only the first three deletion probabilities and first four insertion probabilities have supporting empirical data. Given the rapid decrease in the *A* and *B* deletion parameters, it was assumed that four or more additional deletions would occur with near zero probability. However, a similar assumption could not be made for the insertion probabilities. As will be shown later, it is the occasional low probability for a large number of insertions in a given stream that is mainly responsible for the details of the high *LSF* characteristics of the data set. The *A* insertion parameter is close to 0 after two insertions, and the *B* insertion parameter is approximately decreasing by a factor of 1.3 as the number of insertions increases. Therefore the initial approximation for the probability for *I ≥ 5* insertions occurring was modeled as:

$$P\left(LSF,I\geq 5\right)=0+B_{4-INSERTIONS}\left(1–1/LSF\right)/1.3^{\left(I-4\right)}$$ (9)

where the *B*_*4-INSERTIONS*_ parameter was set as *6*.*2* from Table 1.

#### DTW distance and SNR_Z-NORM_ characteristics

The mean *DTW* distance between the gold-standard and the total *Enolase* data ensemble, Frechet measure [9], was determined as 463 ± 65 across the cleaned squiggles.

The mean *SNR*_*Z-NORM*_ was estimated as 3.8 ± 0.4 for the *SG1600-1699* squiggle grouping using Eq (5). This estimate was based on a value *K = 1*.*25* calculated on the assumption that the number of residual insertions and deletions in *DIFF*_*P-Q*_*(n)* was proportional to the mean *LSF* for this data group

K=squigglemeanlength(1650)/gold−standardlength(1310) (10)

### Poor fit associated with PDF models assuming local distortions

The simple distortion model in Chan et al. [6] assumed that each base in every *DNA* sequence had an equivalent 70% to 90% probability of being correctly represented as a squiggle event, and a 5% to 15% probability of either being duplicated or deleted.

Two variants of this model would match the experimentally observed *x1*.*61* to *x1*.*7* event to base ratios. Either each individual base has a high probability of *P(INSERT) =* 70% of individual duplication, giving a mean *LSF = 1 + P(INSERT)/100*, or a lower *P(INSERT) =* 40% probability of repeated duplications occurring, leading to a mean *LSF = 1 / (1 –P(INSERT)/100);* a better match to the general characteristics of the experimental data.

**Fig 5:**
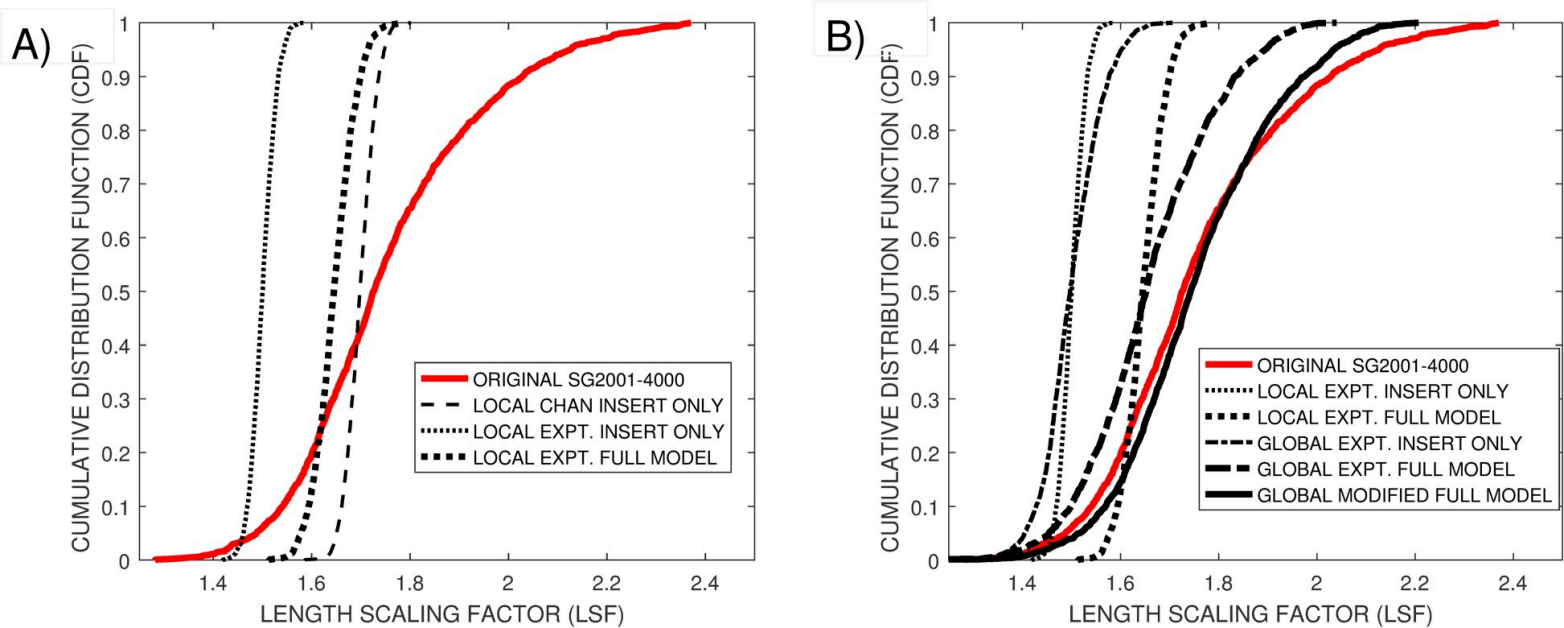
Comparison of original Enolase *SG2001–4000 E-CDF* (red line) against *M-CDF’s* generated using experimental insertion and deletion probabilities applied to A) base-specific and B) squiggle specific simulation models.

In Fig 5A the experimental *E-CDF*, red line, is compared to the *M-CDF* for this simple duplication model, dashed black line. On the hypothesis that there is an underlying model that represents all squiggle distortions in a similar manner in all studies, the *CDFs* are plotted against the length scaling factor, *LSF*, of the squiggles.

Updating this simple model to further account for an *X%* probability that a deletion will occur would require that duplication probability rates be increased to *P(INSERT) = (40 + X)%* to maintain the overall *x1*.*7* event to base ratio. This model was not explored in detail as inclusion of these additional model parameters in the simulation did not provide the wide range of stream lengths that was experimental observed.

A similar narrow *M-CDF*, black dotted line, is generated when the experimental insertion probability rates given in Table 1 are applied to identify introduced distortions as individual bases pass through the nanopore. This model generates a mean *LSF* factor of around *1*.*4*, smaller than the *x1*.*7* found experimentally. Using the full experimental insertion and deletion rates, thick dotted line, increases the mean *LSF* but not the overall length range of the squiggles which remains narrow. The DTW Insertion–Deletion proxies showed that the *1–5* insertion and *1–3* deletion probabilities calculated from these mocked squiggles were lower than found experimentally, Table 2.

**Table 2:** Comparison of insertion and deletion probabilities between the original *SG2001-4000* squiggle ensemble with local and global models using experimental probability rates.

	INSERTION PROBABILITIES^1^						DELETION PROBABILITIES^2^			
	0	1	2	3 s	4	5	0	1	2	3
**Original** ***SG2001-4000***	**66.2±3.8**	**17.1±1.4**	**7.5±1.1**	**3.6±0.8**	**2.0±0.6**	**1.1±0.4**	**96.3±1.0**	**3.3±0.9**	**0.3±0.1**	**0.1±0.1**
***LOCAL MODELS***										
***Modified Chan***	**59.1±1.3**	**23.9±1.2**	**9.9±0.8**	**4.1±0.6**	**1.7±0.4**	**0.7±0.2**	**100±0.0**	**0.0±0.0**	**0.0±0.0**	0.0±0.0
***Experimental*. *Insertion Only***	**70.3±1.7**	**16.7±1.0**	**7.4±0.7**	**1.9±0.4**	**3.5±0.5**	**0.0±0.0**	**100±0.0**	**0.0±0.1**	**0.0±0.0**	0.0±0.0
***Experimental*. *Insertion + Deletions***	**68.9±1.3**	**15.5±1.0**	**6.8±0.7**	**3.3±0.5**	**1.8±0.4**	**1.4±0.3**	**98.3±0.3**	**1.4±0.3**	**0.1±0.0**	0.0±0.0
***GLOBAL MODELS***										
***Expt*. *Insertions Only***	**70.3±2.7**	**16.7±1.3**	**7.4±1.0**	**3.5±0.7**	**1.9±0.5**	**0.0±0.0**	**100±0.0**	**0.0±0.0**	**0.0±0.0**	0.0±0.0
***Expt*. *Insertions + Deletions***	**68.6±3.9**	**15.6±1.5**	**6.9±1.1**	**3.4±0.8**	**1.8±0.5**	**1.4±0.4**	**97.0±0.5**	**2.6±0.5**	**0.4±0.1**	0.0±0.0
***P(INSERT)*** ***= 100 (1–1 / LSF)***	**58.1±5.9**	**23.7±1.5**	**10.1±1.9**	**4.4±1.4**	**2.0±0.9**	**0.9±0.6**	**100±0.0**	**0.0±0.0**	**0.0±0.0**	0.0±0.0
***P(INSERT)*** ***= 100 (1–1 / LSF)*** ***P(DELETE)*** ***= 25*.*5 (1–1 / LSF)***	**65.3±4.5**	**19.7±1.3**	**8.4±1.5**	**3.7±1.2**	**1.6±0.8**	**0.7±0.5**	**96.3±0.5**	**3.3±0.5**	**0.3±0.1**	0.1±0.1
***P(INSERT)*** ***= 114*.*4 (1–1/LSF)*** ***P(DELETE)*** ***= 25*.*5 (1–1 LSF)***	**59.8 ±5.0**	**20.1±1.3**	**9.7±1.5**	**4.9±1.4**	**2.5±1.1**	**1.3±0.9**	**97.1±0.5**	**2.6±0.5**	**0.3±0.1**	0.0±0.0
***P(INSERT)*** ***= 8*.*6+100 (1–1/LSF)*** ***P(DELETE)*** ***= 8*.*7+15 (1–1/LSF)***	**60.7±4.5**	**18.8±1.3**	**9.7±1.4**	**5.0±1.2**	**2.1±1.0**	**1.4±0.7**	**96.2±0.8**	**3.3±0.7**	**0.4±0.2**	0.1±0.1

^1^The empirical insertion probabilities were chosen to better match the experimental and mocked *CDF*

^2^The empirical deletion probabilities were chosen to match those of the original *SG2001-4000* ensemble

### Improved PDF models assuming squiggle-specific distortions

The earlier results we have presented suggest that it is inappropriate to use a simulation model with common probabilities arising from the raw current signals generated as nucleotides pass through nanopores are segmented into individual squiggle events. We suggest an alternate simulation model be considered where there are common insertion and distortion probabilities associated with the entire length of a given raw current signal as it is segmented into a squiggle. The *gev*, log-normal and log-logistic curves provide a reasonable representation of the squiggle *PDF*. We therefore suggest that such common probabilities are associated with errors introduced by either or both of the twin manufacturing processes of producing the RNA molecules to be sampled and the generation of the nanopore sensors rather than being solely by artefacts of an imperfect segmentation process.

#### Squiggle-specific length model based on experimental parameters

Applying this new probability model using the Table 1 parameters associated with specific raw current / squiggle data streams leads to the *M-CDF* shown as the thin dot-dashed line in Fig 5B. This squiggle-specific distortion produces a wider mock *M-CDF*, closer to the width of the empirical *E-CDF*, although having the equivalent mean *LSF* as the local distortion model using the same experimental insertion and / or deletion probabilities,

The introduction of multiple additional insertions

$$P\left(LSF,\;I\geq 5\right)=0+B_{4-INSERTIONS}\left(1–1/LSF\right)/1.3^{\left(I-4\right)}$$ (11)

leads to an even wider *M-CDF*, thick dot-dashed line, that is an improved approximation of the true *E-CDF*. Increasing the scaling factor for insertion rates above 4 from *1*.*3*^*(I-4)*^to *2*.*0*^*(I-4)*^, thick solid line, provides a better fit to the *E-CDF* at high *LSF* values, but begins to over-fit at low *LSF* values. Despite its improved *M-CDF*, we consider it inappropriate to adopt this experimental-based model with its increased scaling factor given the experimental inaccuracies associated with Table 1 parameters.

#### Squiggle-specific length model based on empirical parameters

Based on the experimental results shown in Table 1, we next investigated theoretical insertion and deletion models of the form

$$P\left(INSERT\right)=I_{0}+I_{1}*\left(1–1/LSF\right);\;P\left(DELETE\right)=D_{0}+D_{1}*\left(1–1/LSF\right)$$ (12)

**Fig 6:**
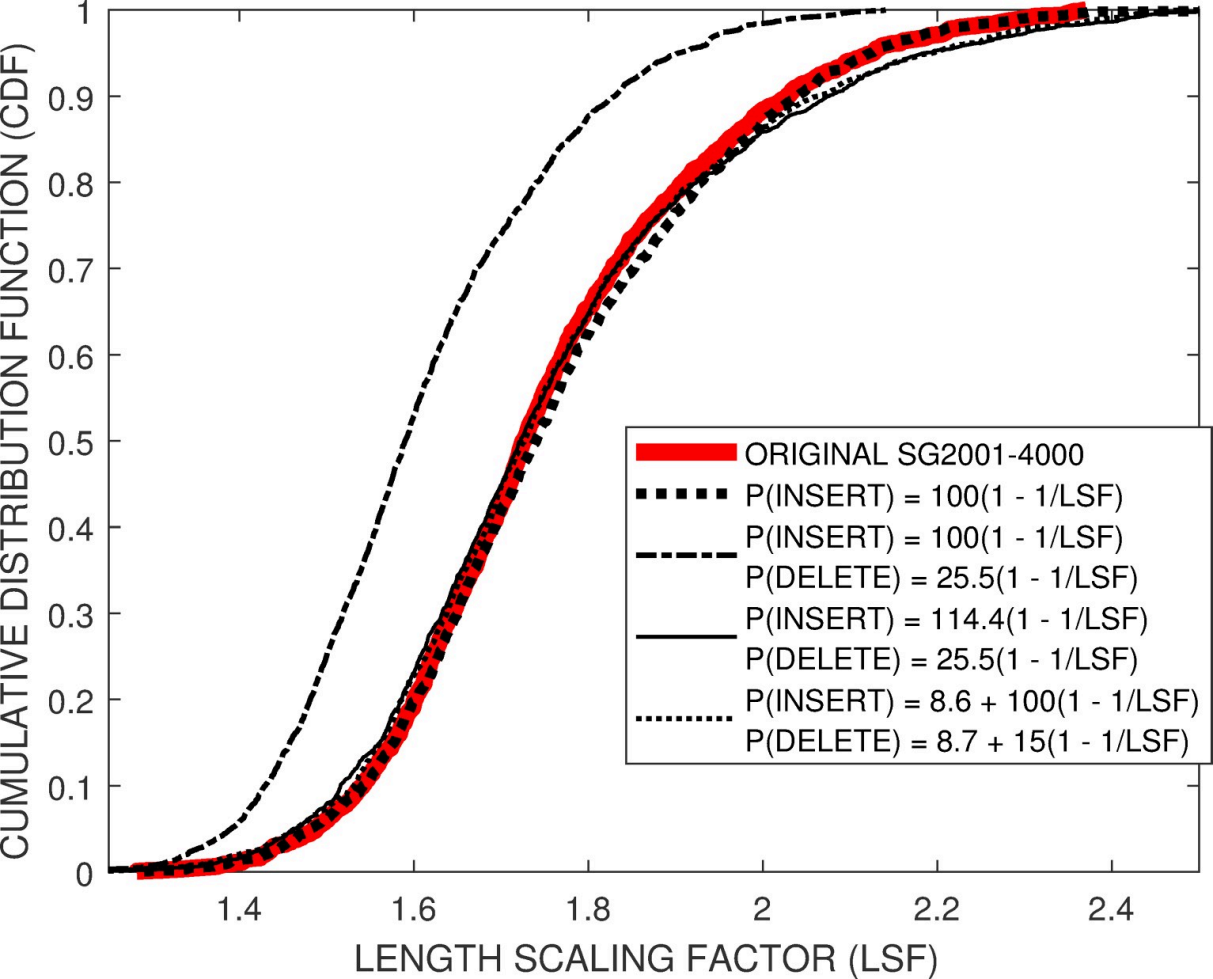
Comparison of the original Enolase *SG2001–4000 CDF* (red line) against *CDF’s* from proposed theoretical insertion and deletion rate models.

The effectiveness of the model would be evaluated by the level of matching between *M-CDF* and *E-CDF*, Fig 6, the level of matching between the insertions and deletion probabilities evaluated by the proposed *DTW* insertion-deletion proxies, Table 2, and the fit to three distributions with long tails, Table 3.

**Table 3:** Comparison of the fits of the original and mocked probability density functions to the long-tailed loglogistic, lognormal and generalized extreme value distributions.

	Loglogistic	Lognormal	Generalized Extreme Value (GEV)
**ORIGINAL**	**mu = 7.73;** **[7.72, 7.74]** **sigma = 0.061;** **[0.058, 0.063]**	**mu = 7.73;** **[7.73, 7.74]** **sigma = 0.106;** **[0.103, 0.111]**	**mu = 2194;[2183, 2206]** **sigma = 217; [209, 225]** **k = -0.10; [-0.13, -0.06]**
**GLOBAL MODELS**			
P(INSERT) = 100 (1–1 / LSF)	mu = 7.73; [7.72, 7.73] sigma = 0.060; [0.058, 0.063]	mu = 7.73; [7.72, 7.74] sigma = 0.106; [0.102, 0.110]	mu = 2190; [2179, 2202] sigma = 212; [204, 220] k = -0.08; [-0.11, -0.04]
P(INSERT) = 114.4 (1–1 / LSF) P(DELETE) = 25.5 (1 –LSF)	mu = 7.73; [7.72, 7.73] sigma = 0.068; [0.065 0.071]	mu = 7.74; [7.73, 7.74] sigma = 0.120; [0.116, 0.124]	mu = 2180; [2167, 2193] sigma = 232; [223, 241] k = -0.023; [-0.06, 0.01]
P(INSERT) = 8 + 100 (1–1 / LSF) P(DELETE) = 8 + 14 (1 –LSF)	mu = 7.74; [7.73, 7.74] sigma = 0.069; [0.066, 0.071]	mu = 7.74; [7.74, 7.75] sigma = 0.121; [0.117, 0.125]	mu = 2187; [2174, 2200] sigma = 236; [227, 246] k = -0.08; [-0.11, -0.05]
Experimental insertion and deletion probabilities	mu = 7.68; [7.68, 7.68] sigma = 0.044; [0.043, 0.047]	mu = 7.68; [7.68, 7.69] sigma = 0.078; [0.075, 0.080]	mu = 2111; [2102, 2120] sigma = 165; [158, 171] k = -0.24; [-0.27, -0.21]
**LOCAL MODEL**			
Experimental insertion and deletion probabilities	mu = 7.68; [7.67, 7.67] sigma = 0.014; [0.013, 0.014]	mu = 7.68; 7.67, 7.68] sigma = 0.024; [0.023, 0.026]	mu = 2136; [2134, 2139] sigma = 52; [50, 54] k = -0.24; [-0.26, -0.22]

There was a near perfect agreement between *E-CDF* and *M-CFD* for *P(INSERT) = 100 (1–1 / LSF)* and *P(DELETE) = 0*, dotted line in Fig 5B. However this model was considered inappropriate as Table 2 shows that applying the *DTW* insertion-deletion proxies to this mocked data provides over-estimated global insertion probabilities in addition to providing zero global deletion probabilities.

However, given that this simple insertion model, *I*_*1*_ * (1–1 / LSF), led to an excellent *M-CFD* approximation, we explored models where both insertion and deletion probabilities having a similar format. Setting *P(DELETE) = 25*.*5 (1–1 / LSF)* restores the deletion probabilities in the mocked data closer to the original experimental values, Table 2. However, this results in a *M-CFD* with a low median value (dot-dashed line). Compensating for the deleted values by increasing the insert probability, *P(INSERT) = 114*.*4 (1–1 / LSF)*, restores *M-CDF* closer to *E-CDF*, thin solid line. However the *DTW* insertion-deletion probes show elevated insertion probabilities compared to the original data, Table 2.

An alternative approach investigated was to set an initial deletion probability to experimental values that approximated those shown in Table 1, *P(DELETE) ~ 9*.*7+ 15 (1–1 / LSF)*, and adjusting *P(INSERT)* to create a match between *E-CDF* and *M-CFD*. Setting *P(DELETE) = 8*.*7+ 15 (1–1 / LSF)* and *P(INSERT) = 8*.*6 + 10 (1–1 / LSF)* gave an *M-CDF* fit, light dashed line, which essentially overlaps the dot-dashed line associated with the previous *P(DELETE) = 25*.*5 (1–1 / LSF)* and *P(INSERT) = 114*.*4 (1–1 / LSF)*.

Both previous models use a compensatory increase in *P(INSERT)* to avoid a low mean *LSF* arising from deletions. An alternative approach of maintaining the length is to consider that the *ME* additional terms in the *WP*_*SQUIGGLE*_*(n)* in Eq (6) are associated with *ME* distortions in the mocked squiggle rather than *ME* deleted terms. While this leads to higher mean *LSF* values without requiring a boosted *P(INSERT)*, the current proposed format of this distortion model is inappropriate as deletion probabilities detected by the *DTW* insertion-deletion proxies now fall to zero.

Table 3 provides an alternative quantitative viewpoint on how well the characteristics match between the original and mocked data streams. The original and mocked data sets were modeled using three distributions with long tails–*log-logistic*, *log-normal* and the *generalized extreme value (gev)*.

- Both the base-specific and squiggle-specific models based entirely on the experimental insertion and deletion rates from Table 1 show low *mu* and *sigma* values for all three distributions reflecting the experimentally unrealistic low mean and narrow range of *LSF* values shown in Fig 5A and 5B.
- The *mu* values describing mean *LSF* characteristics from these three distributions overlap, within experimental error, for all models using empirically determined insertion and deletion probabilities, indicating a general agreement between the original and mocked squiggles. However including the deletion terms in the empirical models leads to higher *sigma* reflecting the miss-matched extreme *LSF* values in the tails of the mocked data shown in Fig 6.

### Matching *DTW* distance and *SNR*_*Z-NORM*_ characteristics

Schultz and Jain [9] applied the Frechet measure to determine the effectiveness of *DTW-*based averaging algorithms that generate a consensus signal from time-distorted signals from multiple sources. In this paper, we propose modifying that measure to provide a second quantitative measure of whether the distortions in the mocked and original data streams are similar.

Table 4 compares the mean and standard deviations of the *DTW* distance between the known gold standard and *N* original and mocked data sets.

$$DTW\_\mathrm{distance}=\sum_{n}dtw(gold,nanostream(n))/N$$ (13)

**Table 4:** Comparing the *DTW* Frechet distance between the original and mocked squiggles and the gold standard. As the complexity of the model increases, the Frechet distance becomes closer between the original and mocked data sets.

	Mean DTW Distance
**ORIGINAL SQUIGGLE**	**483 ± 53**
**LOCAL MODELS**	
Experimental insertions only	27 ± 18
Experimental full model (insertions and deletions)	76 ± 28
**GLOBAL MODELS**	
Experimental Insertions only	29 ± 17
Experimental full model (insertions and deletions)	70 ± 28
P(INSERT) = 100 (1–1 / LSF)	35 ± 23
P(INSERT) = 100 (1–1 / LSF) P(DELETE) = 25.5 (1 –LSF)	109 ± 28
P(INSERT) = 114.4 (1–1 / LSF) P(DELETE) = 25.5 (1 –LSF)	117 ± 34
P(INSERT) = 8.6 + 100 (1–1 / LSF) P(DELETE) = 8.7 + 15 (1 –LSF)	149 ± 34

The similarity between the Frechet distances of the original and mocked squiggles increases with the complexity of the simulation model. We propose that the majority of the remaining differences can be explained in terms of the noise level present on the experimental squiggles.

The mean experimental *SNR*_*Z-NORM*_ was estimated as 3.8 ± 0.4. Table 5 shows the changes in the Frechet distance as the *SNR*_*Z-NORM*_ level changes for the two proposed simulation models. Adding noise to the mocked data set equivalent to *SNR*_*Z-NORM*_ ~ 3.7 to 4.0, **shown in bold**, generated mean and standard deviation of the *DTW* distances between the gold standard and mocked squiggles equivalent to that between the gold standard and original squiggles.

**Table 5:** Comparison of the *DTW* Frechet distance between the original and mocked squiggles and the gold standard as the simulated *SNR*_*Z-NORM*_ *level* deteriorates.

	P(INSERT) = 114.4 (1–1 / LSF) P(DELETE) = 25.5 (1–1 / LSF)	P(INSERT) = 8.6 + 100 (1–1 / LSF) P(DELETE) = 8.7 + 15 (1–1 / LSF)
No noise	116 ± 33	149 ± 34
*SNR*_*Z-NORM*_ = 50	128 ± 30	160 ± 28
*SNR*_*Z-NORM*_ = 20	169 ± 27	199 ± 24
*SNR*_*Z-NORM*_ = 10	247 ± 33	275 ± 29
*SNR*_*Z-NORM*_ = 5	397 ± 50	421 ± 43
***SNR***_***Z-NORM***_ **= 4.0**	**460 ± 55**	**480 ± 50**
***SNR***_***Z-NORM***_ **= 3.7**	**494 ± 60**	**512 ± 54**
*SNR*_*Z-NORM*_ = 3	573 ± 69	588 ± 63
***Original*** ***Frechet distance***	**483 ± 53**	

### Application of the simulation model to other squiggle sets

A final validation of the proposed models was their application to other squiggle sets.

**Fig 7:**
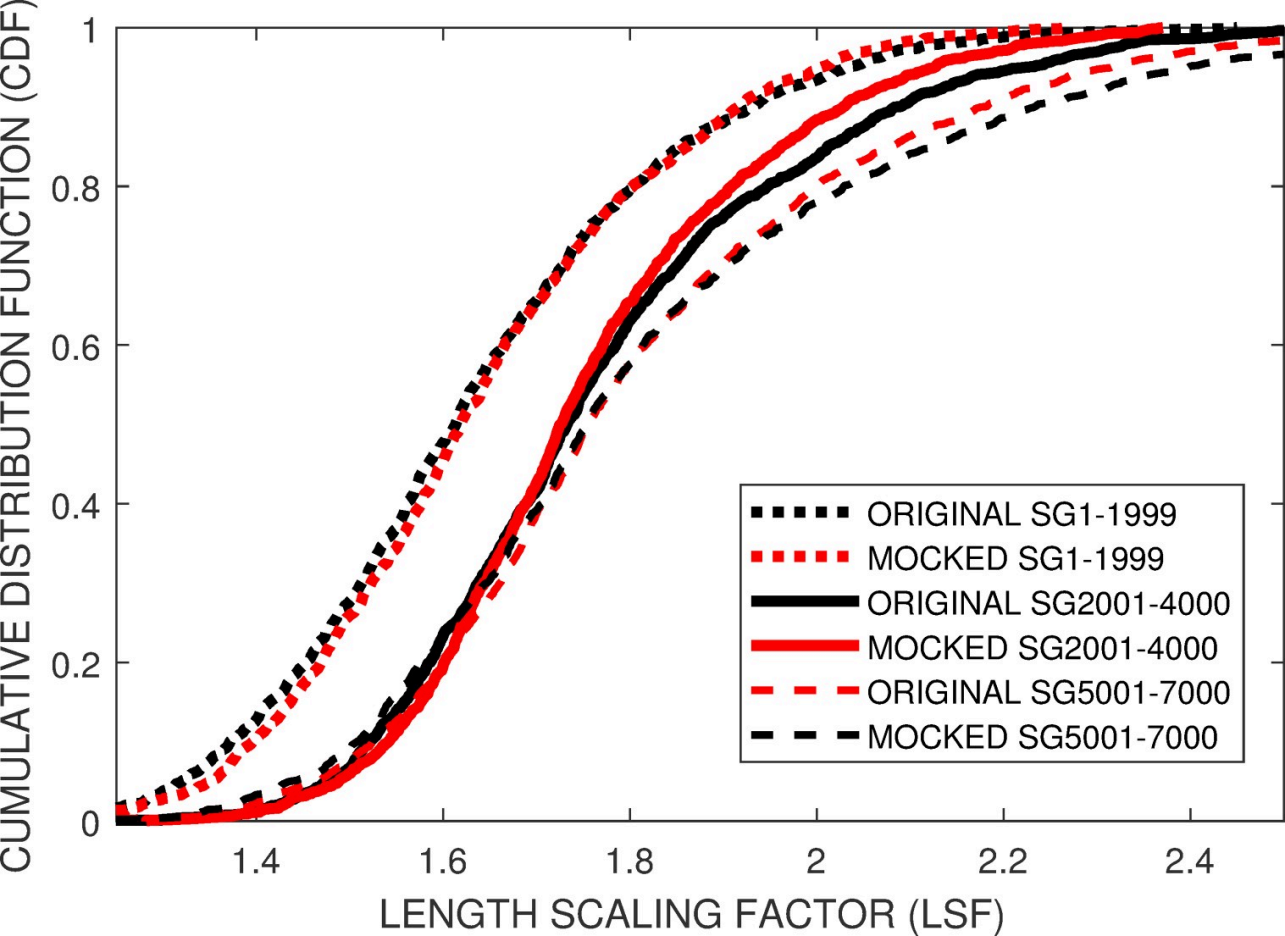
The proposed simulation model provides individual matches between the *E-CDF* and *M-CDF* of the three *Enolase* data streams, *SG1-2000*, *SG2001-4000* and *SG5001-7000*. However additional simulation terms need to be introduced to model the experimental reason behind the increased mean *LSF* value for the *SG2001–4000* and *SG5001-7000 Enolase* streams compared to the *Enolase SG1-2000* and to the *Sequin* squiggles.

**Fig 8:**
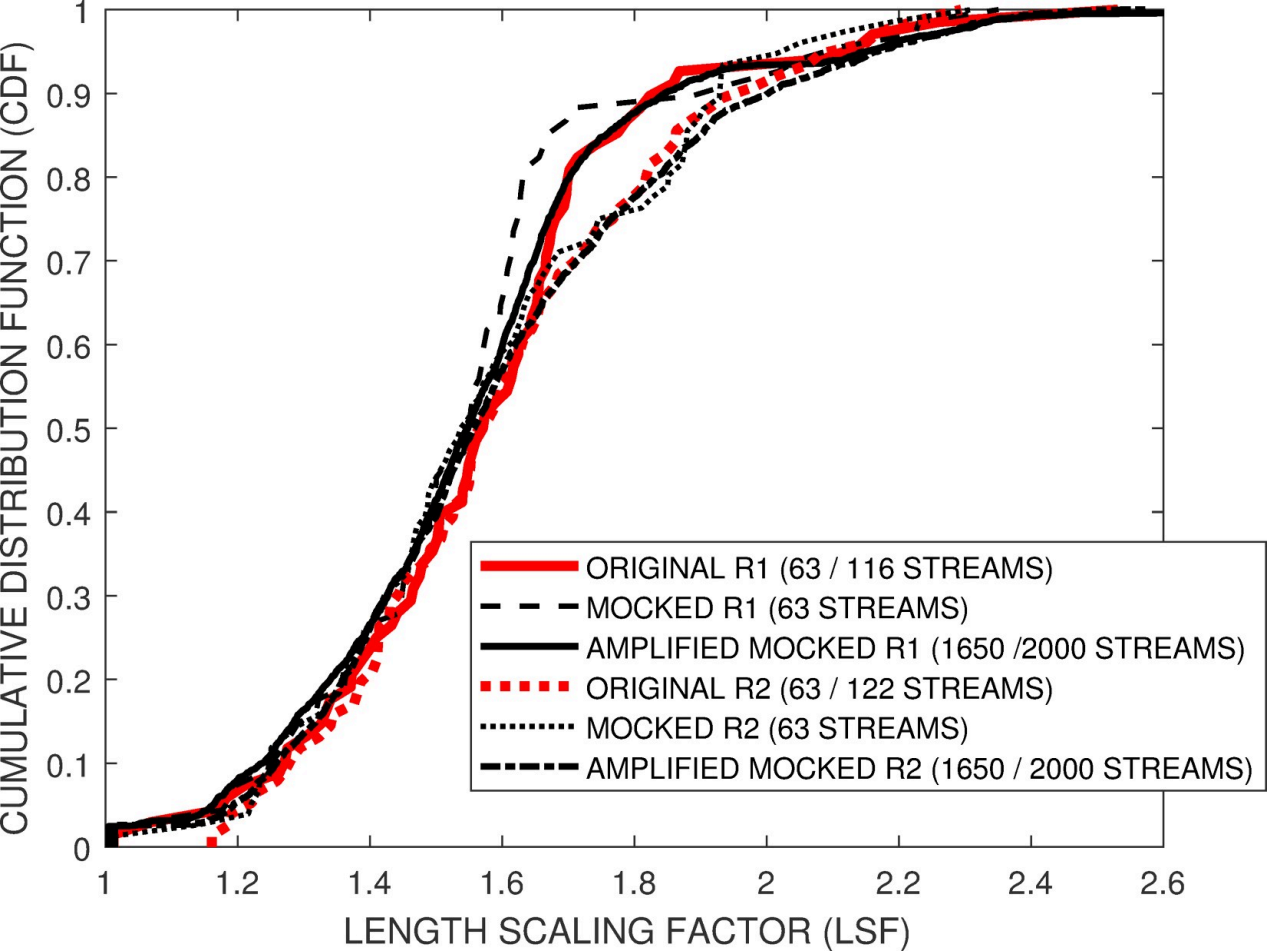
The simulation model provides comparable *E-CDF* and *M-CDF* for the noisier *SEQUIN R1_71_1* and *SEQUIN R 2_55_3* squiggles. Given the low number of experimental available data streams, 116 and 122 respectively, the simulation model is used to generate 2000 mocked streams to provide a smoother *M-CDF* to compare to the original *E-CDF*.

- Fig 7 compares the *E-CDF* of three Enalose squiggle groups, *SG1-2000*, *SG2001-4000* and *SG5001-7000* with the *M-CDF* of the mocked squiggles. It can be seen that there is a temporally-related shift in the mean LSF between the first *SG1-2000* and last *SG5001-7000* group within the *Enolase* squiggle ensemble.
- Fig 8 compares the *E-CDF* and *M-CDF* for the noisier *Sequin R1_71_1* and *Sequin R2_55_3* squiggles, c.f. Fig 1A and 1B. After leader removal and general data cleaning, these sequences respectively provided approximately 65 streams out of the original 116 and 122 squiggles. *M-CDF’s* are shown for simulation models using the equivalent number of squiggles, 65, and using the proposed model and empirical *E-CDF* of these Sequin ensembles to generate a smoother *M-CDF* from 2000 simulated streams.

While differences can be seen, there were comparable fits between the *E-CDF* and *M-CDF* across all five experimental squiggle ensembles (Figs 7 and 8). We therefore conclude that either the proposed insertion and deletion probabilities *P*(*INSERT*) = 114.4 (1−1/*LSF*) with *P*(*DELETE*) = 25.5 (1−1/*LSF*) or *P*(*INSERT*) = 8.6+100 (1−1/*LSF*) with *P*(*DELETE*) = 8.7+15 (1−1/*LSF*) will be appropriate to generate simulated, distorted squiggles within our proposed framework. Appropriate distributions of simulated squiggles *LSF*s can be derived from the *Enolase*-derived *E-CDFs* provided in the depository [16], or *E-CDFs* derived from the user’s own empirical results.

## Conclusion

We have undertaken an investigation of simulation models to characterize the typical distortions introduced by production of raw current signals, and their segmentation into current step level, squiggles, given the stochastic nature of the motor protein, current measurement uncertainty and other factors. This model could be used to assist in optimizing segmentation and squiggle consensus building methods by researchers interested in underlying squiggle space and experimental preparation characteristics, including identifying white-box approaches to identify common distortions, rather than relying on commonly used black box neural network techniques for basecalling nanopore signals.

The underlying characteristics of experimental *Enolase* squiggles produced using an Oxford MinION nanopore-based sequencer and provided squiggle converter were determined. The distribution function of the squiggle length was identified, and new tools proposed to evaluate the probabilities for insertion and deletion distortions introduced as raw current signals are generated and segmented into squiggles. Several simulation models were evaluated by comparing mocked and experimental squiggle characteristics generated from *Enolase* and *Sequin* studies.

A number of common underlying squiggle features were identified while generating the simulation models. A new z-normalized signal-to-noise approach allowed estimation of the noise on the squiggles (~3.7:1 for our RNA datasets). The ratio of the mean-length of the squiggles to the underlying gold-standard length, squiggle event / bases called, was *x1*.*6 –x 1*.*7* across all three studies. Each squiggle ensemble studied was represented by similar cumulative distribution functions, *CDF*. We suggest that the consistent event to base ratio and common *CDF* factors are more than common artefacts produced by an imperfect segmentation algorithm.

The best match between the experimental and mocked data sets occurred if it was assumed that insertion and deletion distortion probabilities describing properties of the segmented squiggle events are related to the properties of the entire (highly variable length) original raw current streams rather than with probabilities associated with individual bases passing through specific nanopores. We speculated that such common global properties might be at least partially associated with variability in the manufacturing processes of physically producing the *RNA* molecules and nanopore sensors and the stochastic nature of the motor proteins rather than improper segmentation of raw current events into individual squiggle events.

There was an unanticipated finding that matches for certain squiggle characteristics were best described using a simulation model involving duplicated insertions of the calculated gold standard signal into the mocked data stream, but no deletions. While this model led to an excellent match between the experimental and mocked CDFs, it led to a poor match of the experimentally determined insert and deletion probabilities. One possible explanation for this observation is that insertions and deletions are generated by different aspects of the distortions introduced by nanopore sequencing devices and / or associated segmentation methods. Further work is under-investigation on how best to employ this and other observations to provide improved experimental and post processing procedures to reduce the impact of distortions on a variety of *DNA / RNA* analyses applied to the raw current or segmented current step level signals.
